# Donepezil can improve daily activities and promote rehabilitation for severe Alzheimer’s patients in long-term care health facilities

**DOI:** 10.1186/s12883-014-0243-7

**Published:** 2014-12-17

**Authors:** Kenichi Meguro, Yoshitaka Ouchi, Kyoko Akanuma, Mitsue Meguro, Mari Kasai

**Affiliations:** Division of Geriatric Behavioral Neurology, CYRIC, Tohoku University, 2-1, Seiryo-machi, Aoba-ku, Sendai, Miyagi 980-8575 Japan

**Keywords:** Alzheimer’s disease, Donepezil, Cholinesterase inhibitors, Life expectancy, Nursing home

## Abstract

**Background:**

Cholinesterase inhibitors can delay the progression of Alzheimer’s disease (AD). Several clinical trials of the drug in *moderate to severe* AD have consistently reported clinically positive effects. A combining effect with psychosocial intervention was reported in *mild to moderate* AD patients. Since a therapeutic approach or rehabilitation combined with cholinesterase inhibitors for severe AD patients remains controversial, we performed a prospective intervention for patients in Long-Term Care Health Facilities (LTCHF).

**Methods:**

Two LTCHFs (N1, N2) were enrolled. N1 is a 126-bed facility that does not treat with donepezil but rather with psychosocial intervention (reality orientation and reminiscence). N2 is a 150-bed facility with a 50-bed special dementia unit, in which the physician can prescribe donepezil. On top of the similar psychosocial intervention, rehabilitation is performed in N2. Thirty-two severe AD patients (MMSE < 6) in N1 and N2 (16 vs. 16) were compared for the effect of donepezil (10 mg/d for 3 months) with or without psychosocial intervention (n = 8 vs. 8 for each facility). The Vitality Index was used to assess daily activities and the introduction of rehabilitation.

**Results:**

The response ratio (MMSE 3+) of donepezil was 37.5% in N2. The combination of donepezil with the psychosocial intervention improved the Vitality Index total score, and Communication, Eating, and Rehabilitation subscores (Wilcoxon, p = 0.016, 0.038, 0.023, and 0.011, respectively). Most of them were smoothly introduced to rehabilitation, and the proportion of accidental falls decreased. Psychosocial intervention in N1 without the drug only improved the total score (Wilcoxon, p = 0.046).

**Conclusions:**

A combined therapeutic approach of donepezil and psychosocial intervention can have a positive effect, even for severe patients through the introduction of rehabilitation and decreasing accidental falls. However, these findings require replication in a larger cohort.

## Background

The number of Alzheimer’s disease (AD) patients continues to increase worldwide and thus the establishment of an appropriate therapeutic approach including drug, psychosocial intervention, and rehabilitation is necessary. For drug treatment, cholinesterase inhibitors (ChEIs), such as donepezil, have clinically positive effects, leading to improvements in cognitive function for a certain duration and delaying the progression of the disease [1]. The drug can delay the deterioration of daily function [2], thus decreasing the caregivers’ burden [3]. Indeed, the severity of the daily functional disability is the most critical factor in keeping AD patients living in their homes prior to placement in nursing homes [4].

Most AD patients residing in nursing homes have severe stage of disease characterized by a profound cognitive loss as well as deterioration in functional abilities [5]. Thus, the treatment of severe AD is expected to improve or stabilize such symptoms [6,7]. Several clinical trials of ChEIs in moderate to severe AD [8-11] have consistently reported such effects. Jelic et al. [12] studied the efficacy of donepezil for severe AD patients in 248 nursing homes, and demonstrated that donepezil treatment showed stabilization or improvement across multiple outcome measures.

Since ChEIs are not “curative” drugs, the addition of psychosocial interventions are also considered in order to optimize the function of patients. The reminiscence approach [13] seeks to facilitate the recall of past experiences and thereby improve well-being or quality of life (QOL). Relatively well preserved remote memory in AD [14] can provide a neurological basis to support this approach. The reality orientation (RO) technique [13,15] is also used, which stimulates time and place orientation, usually by providing information regarding orientation. It is usually used in combination with the reminiscence approach [16,17].

We have previously reported a positive effect of combined donepezil and psychosocial intervention, including the RO with the reminiscence approach, on the patients’ QOL [18]. However, such positive effects of the drug are not always recognized by the patients and caregivers; thus, leading them to discontinue the drug treatment easily [19]. Umegaki et al. [20] surveyed the persistence of donepezil therapy and found that only 50% of AD patients remained on treatment after one year. The main reason for drug withdrawal was the perceived “ineffectiveness” of the drugs by the patients and caregivers, which may contrast with their unrealistic expectations, such as “improving” memory function.

Although benefit of psychosocial intervention such as RO and physical reactivation program in combination with donepezil for mild to moderate AD has been reported [21], no such studies have been performed for patients with severe stage of disease. Such a combined therapeutic approach especially for severe AD patients would be better performed at the institutes, since caregivers are also getting old and even suffering from mild cognitive impairment in Japan, and high-quality psychosocial intervention is not frequently possible at home.

According to the Long-Term Care Insurance, three kinds of institutes are available in Japan, i.e., the Long-Term Care Health Facilities (LTCHF), Special Elderly Nursing Homes, and Group Homes. The situations of drug treatment are different: patients living in the latter two institutes are able to consult doctors at other clinics to receive prescriptions. However, after placement in the LTCHF, by law the patients can only take medicines provided by the institute, and unfortunately expensive drugs, such as ChEIs, are not always provided. Managers of the LTCHFs decide independently whether or not the ChEIs are provided for economic reasons. We believe that this is a second reason for drug withdrawal [19].

However, we actually experienced patients with severe AD who experienced a positive effect by the combination of donepezil and psychosocial intervention. It may be due to the introduction of rehabilitation that was reported to improve ADL. The aim of the study is to investigate whether ChEIs together with psychosocial interventions have clinically positive effects for patients with severe AD. We hypothesized that even for LTCHF-replacement patients with severe AD, ChEIs together with psychosocial interventions have such effects. This effect included improving daily activities and promoting rehabilitation, as well as decreasing the incidence of accidental fall. Data was collected prospectively from the two types of LTHCFs.

## Methods

### LTCHF

Two LTCHFs (N1, N2), which are located in Miyagi Prefecture, Northern Japan, were enrolled. They are run by the same company and have a close relationship with our research group. N1 is a 126-bed facility without donepezil but psychosocial intervention (reality orientation and reminiscence) is performed. N2 is a 150-bed facility with a 50-bed special dementia unit. After the approval of donepezil (see below), physicians became able to prescribe donepezil at that site. In addition to the similar psychosocial intervention, rehabilitation is performed in N2.

### Patients

Thirty-two severe patients with AD (16 in N1 and 16 in N2) were studied. The inclusion criteria were: 1) to meet the diagnostic criteria for probable AD according to the NINCDS-ADRDA [22] with reference to the CT or MRI images; 2) the scores on the Mini-Mental State Examination (MMSE) [23] were less than 6; 3) more than three months had passed since placement in N1 or N2; and 4) they were all able to walk with or without canes and eat with the help of the staff.

The exclusion criteria were 1) significant neurological signs, such as hemiparesis or swallowing disturbance; 2) the presence of aphasia that prevented them from verbal communication with the staff; 3) severe behavioral symptoms, such as delusion or delirium and wandering, that prevented them from joining psychosocial intervention or rehabilitation; 4) history of stroke, cerebral contusion, or other systematic disorders that could affect central nervous system function, i.e., hypothyroidism or decreases in vitamin B_1_, B_6_, B_12_.

Informed consent was received from the family members of all patients analyzed. This study was approved by the Medical Ethics Committee of Tohoku University and those of LTHCF N1 and N2.

### Psychosocial intervention in N1 and N2

The RO and reminiscence approaches are routinely performed as a group work in N1 and N2. Sixteen patients in N1 and N2 were randomly divided into two arms, i.e., those who received psychosocial intervention (n = 8, each for N1 and N2) and those who received normal care (n = 8, each for N1 and N2). Rehabilitation for walking was also performed by a physical therapist in N2.

The characteristics of N1 and N2 facilities and the information on the patients’ demographic and clinical characteristics the patients studied are shown in Table 1. Baseline characteristics are the same through the groups.

**Table 1:** **Donepezil administration for AD patients in LTCHF (n = 32) +/− Psychosocial Intervention**

		**LTCHF-N1**	**LTCHF-N2**
		126 beds	150 beds
**Dementia ward**		None	50 beds
**Donepezil**		None	Yes
**Subjects**		(16 patients)	(16 patients)
**Usual care**	n	8	8
	M/F	1/7	2/6
	MMSE Mean (SD)	3.6 (1.1)	3.5 (0.9)
**Psychosocial intervention**	contents	RO + Reminiscence	RO + Reminiscence, Rehabilitation
	n	8	8
	M/F	2/6	2/6
	MMSE Mean (SD)	3.6 (1.2)	3.6 (1.4)

AD = Alzheimer’s disease, LTCHF = Long-Term Care Health Facility, RO = Reality Orientation, M = male, F = female, MMSE = Mini Mental Sate Examination.

### The use of Donepezil in N2

After explanation of the study protocol, a manager at N2 agreed to employ the 3 mg, 5 mg, and 10 mg tablets of donepezil. After confirming the absence of side effects in response to 3 mg/day for 1 or 2 weeks, 10 mg/day of donepezil was administered for 16 patients in N2.

### Outcomes

To address the hypothesis described above, the three outcomes below were measured pre- and post-interventions. Post-intervention assessments were performed after a period of 3 months. During this three-month period, the drugs were not changed.

#### Vitality index

The Vitality Index [24] was used to assess daily activities and the introduction of rehabilitation. This index is established and validated by Toba et al., comprised of five items, with each of them being assessed according to 3 ratings, as follows: 1) Waking pattern (2: organized pattern of waking, 1: requires a caregiver’s aid occasionally, 0: Never wakes voluntarily), 2) Communication (2: vocalizes reciprocal exchanges at will, 1: responsive to verbal stimulation, 0: no cognitive response), 3) Feeding (2: motivated to eat, 1: passive, but eats with encouragement, 0: indifferent to eating), 4) On and off toilet (2: independent or never fails to express micturition desire, 1: does not express micturition desire consistently, 0: indifferent to voiding), 5) Rehabilitation and other activities (2: motivated to be rehabilitated or to be involved in other activities, 1: passive but tries with encouragement, 0: refuses or indifferent). The staffs at the two LTCHFs receive joint uniform training in usage of the scale.

#### MMSE

The MMSE was used to assess general cognitive function.

#### Accidental falls in N2

The records of risk management in N2 before and after donepezil administration were analyzed retrospectively.

### Analyses

#### Analysis 1 (N1): effect of psychosocial intervention without donepezil

Sixteen patients in N1 did not receive donepezil. Eight patients undergoing normal care and 8 patients who underwent the RO and reminiscence approaches were compared to reveal the effect of psychosocial intervention in the absence of donepezil.

#### Analysis 2 (N2): effect of psychosocial intervention combined with donepezil

Sixteen patients in N2 received donepezil. Eight patients undergoing normal care and 8 patients who underwent the RO and reminiscence approaches were compared to reveal the effect of psychosocial intervention combined with donepezil.

#### Analysis 3: accidental falls (N2)

The records of risk management in N2 before and after donepezil administration were analyzed retrospectively. The accidents were categorized to “Fall,” “Bruise,” “Drug,” “Swallow,” “Bed,” and “Loss.” “Fall” herein means fall down on the floor during walking, “bruise” shows any kind of bruise on their bodies, “drug” indicates mal-taking medicines provided for other patients, “swallow” means swallowing something by mistake, “bed” shows any kind of troubles around the beds, and “loss” indicates going out of the institutes by mistake.

Non-parametric Wilcoxon matched-pairs signed-rank tests were performed to reveal pre and post differences for Analyses 1 and 2. The Chi-square test was used for Analysis 3. The significance levels were all set at 0.05. The IBM SPSS Statistics 22.0 was used.

## Results

All 32 patients completed the intervention. None of the patients exhibited acute diseases, such as pneumonia or fever that would warrant excluding the patients from the study protocol.

### Analysis 1 (N1): effect of psychosocial intervention without donepezil

**Figure 1:**
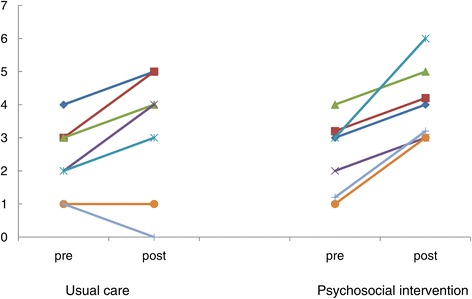
**The normal care group showed no significant changes; however, the psychosocial intervention group exhibited improvement in the total scores (Wilcoxon, p < 0.05).** No significant differences were noted for the Vitality Index subscores. Vitality Index changes for psychosocial intervention in LTCHF-N1.

Figure 1 illustrates the changes of the Vitality Index total scores for psychosocial intervention in N1. The normal care group showed no significant changes; however, the psychosocial intervention group exhibited improvement in the total scores (Wilcoxon, p = 0.046). No significant differences were noted for the Vitality Index subscores.

### Analysis 2 (N2): effect of psychosocial intervention combined with donepezil

**Figure 2:**
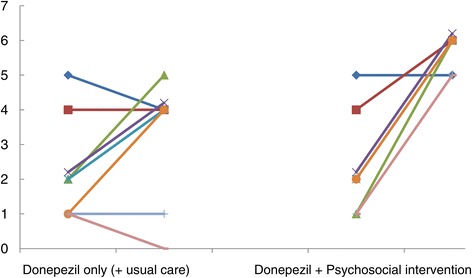
**Similar to N1, the normal care group showed no significant changes; however, the psychosocial intervention group exhibited improvement in the total scores (Wilcoxon, p < 0.05).** The Vitality Index subscores of Communication, Eating, and Rehabilitation were also improved (Wilcoxon, p < 0.05). Vitality Index changes for donepezil with or without psychosocial intervention in LTCHF-N2.

Figure 2 illustrates the changes of the Vitality Index total scores for psychosocial intervention combined with donepezil in N2. Similar to N1, the normal care group showed no significant changes; however, the psychosocial intervention group exhibited improvement in the total scores (Wilcoxon, p = 0.016). The Vitality Index subscores of Communication (p = 0.038), Eating (p = 0.023), and Rehabilitation (p = 0.011) were also improved.

Table 2 notes the changes of MMSE, observed behavior and cognition in 16 patients in N2. The IDs #1 through #8 received donepezil and normal care only, and #9 through 16 received donepezil and psychosocial intervention. Since six patients (IDs#9-11 and #14-16) showed an increase of 3 or more MMSE points during the 3 months follow-up (MMSE 3+ improvement), the response ratio of donepezil was calculated as 37.5% (6/16). Eight patients (IDs #9 through 16) were introduced smoothly to rehabilitation and one patient (ID #9) was discharged from N2 and returned to her home. The case report will be presented below for better understanding of these results.

**Table 2:** **Donepezil administration to AD patients in LTCHF-N2**

**ID**	**MMSE pre**	**Psychosocial Intervention**	**Changes**		**MMSE post**	
			**Behavior**	**Cognition**		
1	5	None	Decreased refusal of being cared	Conversation with the staff	5	
2	5				4	
3	4				4	
4	4				3	
5	3		No changes		3	
6	3				2	
7	3			No changes	2	
8	2				5	
9	5	Done	Promote rehabilitation	Conversation with the staff	9	*
10	5				8	*
11	4				7	*
12	4				5	
13	4				3	
14	3				6	*
15	2				6	*
16	2				5	*

AD = Alzheimer’s disease, LTCHF = Long-Term Care Health Facility, MMSE = Mini-Mental State Examination.

*donepezil responders as shown by MMSE 3 + .

### Case report

The patient (#ID 9 in Table 2) was an 89-year-old woman. She got married when she was 24 years old. Her husband was killed in World War II when she was 25 y. Thereafter, she obtained a hairdresser’s license and ran a barber shop with her nephew. When she was 84 years old, she had difficulty in financial management and closed her barber shop. When she was 86 years old, she had a difficulty in maintaining hygiene in the house. Since her room had become verminous, a public health nurse forced her to be placed in N2. Her MMSE score was 7. After living in N2, she had nothing to do and just spent her time watching TV.

Taking into consideration her past life history, we prepared hair mannequins for her rehabilitation. A session was made up of occupational therapy of hair dressing using hair mannequins, together with reality orientation and the reminiscence approach. We performed one hour per day per week for 3 months. At the beginning, she told us that she had never seen hair mannequins when they were initially presented to her. After administration of donepezil 5 mg/day combined with the rehabilitation, her attention was stimulated. After 2 months, she recalled that she had been a hairdresser and indulged in reminiscence about her past life. She claimed that other patients were lacking in sanitation. Although her MMSE score was not increased to 8, her Vitality Index increased dramatically from 1 to 6, with increases for all subscores. A physical therapist reported that she had become active and had more attention for the rehabilitation program of standing and gait. After three months, she was finally discharged from N2 and returned to her home and started to live together with her nephew.

### Analysis 3: accidental falls in N2

Table 3 notes the records of risk management in N2 before (6 months, April to September 201X) and after (October 201X ~ March 201X + 1) donepezil administration. The exact year was masked by the N2 staff. The number of accidental “Fall” decreased significantly after donepezil administration (chi-square test at 0.05). The staff reported that the patients who had received donepezil had developed more attention to their environment and were more cautious in preventing falls.

**Table 3:** **Risk management before/after donepezil deployment in N2**

	**Before**		**After**	
	**201X.4 ~ 10**	**Mean (Month)**	**201X.10 ~ 201X + 3.**	**Mean (Month)**
**Fall**	83	11.9	38	7.6
**Bruise**	6	0.9	4	0.8
**Drug**	6	0.9	5	1.0
**Swallow**	0	0	1	0.2
**Bed**	2	0.3	0	0
**Loss**	0	0	0	0

## Discussion and consclusions

As described in the introduction, several clinical trials of ChEIs in *moderate to severe* AD have consistently reported clinically positive effects. A combining effect with psychosocial intervention was reported in *mild to moderate* AD patients. We herein performed a combining approach for *severe* AD patients in LTCJFs, and found that a combined therapeutic approach of donepezil and psychosocial intervention can have a positive effect, through the introduction of rehabilitation and decreasing accidental falls.

### Effect of psychosocial intervention

The results in Analysis 1 (N1) demonstrated that psychosocial intervention, including the RO and reminiscence approach, was effective in the absence of donepezil administration. However, the effect was considered to be weaker than that achieved in combination with the drug (Analysis 2 (N2)), since no significant differences were noted in the subscores.

Clinically, we know that AD patients who manifest recent memory deficit can maintain intact remote memory, and that they can retain their skills. We considered the patient’s life history and designed a psychosocial intervention program that was aligned with the patients’ remote memories and skills. Good emotional relationships between the patients and staff, as shown by perfect participation rates, can enhance the positive effect of the intervention content.

### Effect of combined donepezil administration and psychosocial intervention

The results in Analysis 2 (N2) revealed several things. The effects of donepezil on MMSE were not apparent unless the psychosocial intervention was added. This meant that the drug was considered to be “ineffective” according to the MMSE criteria for drug responders. This was probably due to the limitation of the dose of 10 mg/day of the drug, and while the use of 23 mg/day donepezil is anticipated, it is not yet permitted in Japan.

However, when the psychosocial intervention was provided in combination with the drug, the MMSE-based response ratio was calculated as 37.5%. All patients receiving the combined drug and psychosocial interventions (IDs #9 through 16) were introduced smoothly for rehabilitation and one patient (ID #9) was discharged from N2 and returned to her home. Previous reports have indicated that the drug could stimulate attention through the frontal-parietal or basal ganglia networks [25-27]. The preservation of function of the patients, even in the severe stage of AD, was suspected to be activated by psychosocial intervention, after stimulation of the patient’s attention function by donepezil. The decreased rate of falling was also suspected to be due to such a combined effect. These findings also suggest that psychosocial intervention could be considered to be an outcome of the donepezil treatment.

The financial costs of combining of drug and psychosocial intervention might worry LTHCF managers. However, after an effective combining intervention, the ratio of discharge of the patients to their homes might increase like ID #9. This increased “turnover” can obtain additional income by the LTCI.

### Limitation of the study

In this study, we could examine only two LTCHFs. Indeed, it is not easy to involve LTCHFs for research, especially for drug treatment, since it is directly connected to the matter of management. The N1 and N2 facilities have close relationships with our laboratory, and patients there have been able to undergo CT or MRI for the purpose of research. Therefore, we should cautious about the institution bias in interpreting the results. For statistical analyses, we did not perform a three-way design (Institute*drug*psychosocial intervention) due to the limited numbers of patients. Regarding the outcomes, we used the Vitality Index, an observational scale, which is affected by the skill of the observers. However, the Japanese version is standardized and the 3 ratings are clinically available. Also, unlikely to the Severe Impairment Battery (SIB) [28], MMSE score is not an appropriate measure for patients with severe AD. Potential cognitive changes might not have been detected, which can lead to less accurate results. Although the above-noted limitations were present, we believe the results could provide useful information on the therapeutic approach for severe AD patients in LTCHFs.
